# The Effect of Long-Term Traditional Chinese Medicine Treatment on Survival Time of Colorectal Cancer Based on propensity Score Matching: A Retrospective Cohort Study

**DOI:** 10.1155/2020/7023420

**Published:** 2020-01-31

**Authors:** Yuli Wang, Ping Liu, Yuan Fang, Jianhui Tian, Shufang Li, Jing Xu, Fanchen Zhao, Xiaoling Yin, Qiang Zhang, Yan Li

**Affiliations:** ^1^Shanghai Municipal Hospital of Traditional Chinese Medicine, Shanghai University of Traditional Chinese Medicine, Shanghai, China; ^2^Ganquan Street Community Hospital of Shanghai Putuo District, Shanghai, China; ^3^Shanghai Longhua Hospital, Shanghai University of Traditional Chinese Medicine, Shanghai, China; ^4^Shanghai Putuo District Central Hospital, Shanghai University of Traditional Chinese Medicine, Shanghai, China

## Abstract

**Objective:**

To explore the effect of long-term traditional Chinese medicine (TCM) treatment on survival time of colorectal cancer (hereinafter referred to as CRC).

**Methods:**

Our clinical study included patients who were diagnosed with CRC clinically or pathologically. Patients were divided into TCM treatment group and control group according to whether the modified Anti-cancer Decoction II Formula was applied for more than six months. Propensity score matching (hereinafter referred to as PSM) was used to further balance the covariates between groups. One-year to six-year progression-free survival rates of the two groups and the median progression-free survival (mPFS), median overall survival (mOS) of the two groups before and after PSM were calculated respectively. Furthermore, 15 factors that may affect the mPFS in CRC were included in COX multivariate regression analysis to explore the prognostic factors related to CRC as well as to analyze the risk ratio of different subgroups.

**Results:**

A total of 529 CRC patients were included in our study, 285 patients were in the TCM treatment group and 244 patients were in the control group. Before PSM, the mPFS and mOS in the TCM treatment group were 68 months and 75 months respectively, while mPFS and mOS in the control group were 40 months and 65 months respectively. After PSM, mPFS and mOS in the TCM treatment group were both 75 months, while mPFS and mOS in the control group were 28 months and 44 months respectively. One-year to six-year progression-free survival rates were 94.0%, 76.1%, 64.7%, 57.9%, 52.0%, 44.1% respectively in the TCM treatment group, and 78.6%, 61.4%, 51.7%, 40.8%, 33.0%, 29.1% respectively in the control group (*p* < 0.01)_._ COX multivariate regression analysis indicated that surgery, chemotherapy and taking Chinese herbal decoction were protective factors for CRC recurrence and metastasis, while combining with intestinal obstruction, drinking history and family history were independent factors for CRC recurrence and metastasis. The results of subgroup analysis showed that the decoction of TCM could reduce the risk of recurrence and metastasis in each subgroup (*p* < 0.01)_._ COX multivariate regression analysis indicated that surgery, chemotherapy and taking Chinese herbal decoction were protective factors for CRC recurrence and metastasis, while combining with intestinal obstruction, drinking history and family history were independent factors for CRC recurrence and metastasis. The results of subgroup analysis showed that the decoction of TCM could reduce the risk of recurrence and metastasis in each subgroup (

**Conclusions:**

Long-term TCM treatment by the usage of the modified Anti-cancer Decoction II Formula not only has a positive effect on the survival time of CRC patients, but also helps reduce the risk of recurrence and metastasis of CRC, which can be flexibly applied in the whole process of CRC treatment.

## 1. Introduction

CRC is a common gastrointestinal malignancy in China. According to statistical results published by GLOBOCAN 2018 [1], the incidence of CRC worldwide was 10.2%, ranking the third, while the death rate was the second highest at 9.2%, which displayed an upward trend from previous years. With the development of China's national economy and the improvement of social's lifestyle, the mortality of CRC in urban areas is significantly higher than that in rural areas in recent years [2]. Therefore, the prevention and treatment of CRC is facing a severe challenge [3]. Although radical resection is the main and the most effective treatment for CRC presently [4], a considerable number of patients with advanced CRC have little benefit due to poor systemic conditions or even combined with multi-organ metastasis. As a result, the application of combining with a variety of treatment methods, such as chemotherapy, radiotherapy, and biotherapy is imperative. As a diagnosis and treatment method with Chinese characteristics, TCM has become an indispensable treatment method in the comprehensive treatment of CRC due to its unique treatment based on syndrome differentiation and holistic regulation. Studies in recent years have showed that TCM could prolong the survival time of patients with CRC effectively as well as improve the life quality of patients significantly [5]. It can also be combined with chemotherapy to prevent postoperative recurrence and metastasis of CRC [6–8]. Experimental studies have revealed that the TCM compound exerted anti-tumor effect by affecting the proliferation, cell cycle, apoptosis, autophagy, chemotherapy resistance and other multiple pathways and targets of CRC cells [9].

The Anti-cancer Decoction II Formula is a prescription for the treatment of digestive system tumors, which is developed by Professor Yan Li based on more than thirty years clinical experience. This prescription upholds academic thought “strengthen the healthy Qi to anti-cancer” of Jiaxiang Liu, the master of TCM. The Radix Astragali, Codonopsis Pilosula, Poria Cocos and Semen Coicis in this prescription can inhibit tumor growth by improving cellular immunity and humoral immunity to some extent [10–13]. Our previous study showed that Anti-cancer Decoction II Formula could improve the clinical symptoms and the life quality of CRC patients significantly. Additionally, by comparing the changes of immune function before and after the treatment, we found that the prescription could regulate the immune function of patients by down-regulating CD^8+^ T cell [14]. However, there're still few studies about this prescription on the survival of CRC patients.

Therefore, in order to further explore the influence of TCM treatment on the survival of CRC, we conducted a multi-center, large sample retrospective study. Method of PSM is widely used in the situation of imbalanced covariate between groups. In addition to the advantage of large sample size, many high-quality retrospective studies usually use PSM to balance the covariates between the groups, such as Sooriakumaran research [15], Polanco research [16], van Erning's research [17] and so on. Hence, when we analyzed the clinical data, we used PSM so as to scientifically evaluate the efficacy of long-term TCM treatment on the survival of CRC patients.

## 2. Methods

### 2.1. Study Design and Participants

This study was designed as a multicenter, retrospective observational cohort study, which involved Shanghai Municipal Traditional Chinese Medicine Hospital and Central Hospital of Shanghai Putuo District. The study protocol was approved by the Ethics Committee of Shanghai Municipal Traditional Chinese Medicine Hospital. From 2010 to 2018, 580 patients diagnosed with colorectal cancer pathologically or clinically were screened and 529 were included in our study. The patients with the following conditions were excluded from our study: (1) diagnosed as secondary CRC or combined with other organ malignancies; (2) follow up data were severely fragmented; (3) The KPS score was less than 60 points, or combined with other severe basic diseases such as heart, liver, kidney.

**Figure 1:**
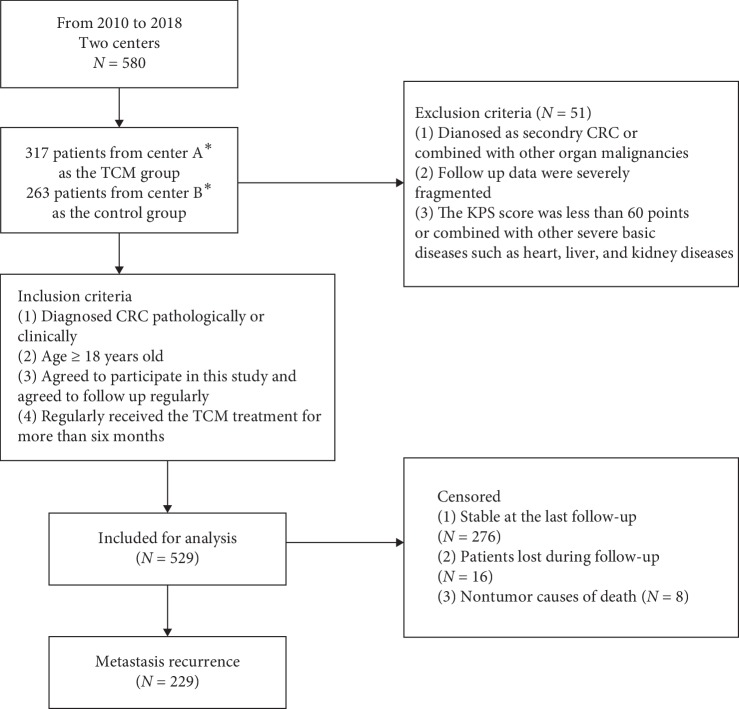
The flow chart of patients' selection and censoring for the study. ^*∗*^Center A Shanghai Municipal Traditional Chinese Medicine Hospital. Center B Shanghai Putuo District Central Hospital.

All of 285 patients who were treated with long-term TCM systematically were divided into the TCM treatment group and 244 patients who were not treated with TCM treatment were divided into the control group (Figure 1).

### 2.2. Long-Term Systematic TCM Treatment

Long-term systematic TCM treatment was defined as the patients who received the TCM treatment regularly, and they were recommended to take Chinese herbal decoction at least six months continuously. All patients in the TCM treatment group took the modified Anti-cancer Decoction II Formula, which was composed of Radix Astragali, Rhizoma Atractylodis, Codonopsis Pilosula, Poria Cocos, Tangerine Peel, Semen Coicis, Dioscorea Opposita, Fructus Lycii, Glossy Privet Fruit, Angelica Sinensis, Rhizoma Polygonati, Oldenlandia Diffusa, Chinaroot Greenbrier, Wild Grape-vine, Radix Actinidiae Chinensis, Rehmannia Glutinosa Libosch, Selfheal, Gecko and Centipede.

Additionally, the dosage and composition of the herbal were adjusted by clinicians according to the specific situation. For example, patients with abdominal distension were treated with the addition of Bitter Orange and Magnolia Bark. For patients with fever and perspiration, Gypsum and Anemarrhena were added. Patients who suffered with constipation were treated with the addition of Rhubarb and Mirabilite. For cancer pain, Szechwan Chinaberry Fruit, Corydalis Tuber, Radix Paeoniae Alba and Liquorice were added. For poor appetite and nausea, Medicated Leaven, Roasted Malt, Bamboo Shavings and Inula Flower were added. Decoct the concentrated herbs to 300 ml, take it in the morning and evening 2 times after meals. The herbs were provided by Chinese medicine pharmacies of Shanghai Municipal Traditional Chinese Medicine Hospital.

### 2.3. Follow up Scheme

#### 2.3.1. Follow up Method

Clinical data was followed up by several researchers routinely each six months at different interval by clinical return visit or phone call. Information such as death time of missing patients was collected from Shanghai Disease Control and Prevention Center.

#### 2.3.2. Follow up Contents

Clinical data included the following data:

1. General information: first visit time, gender, age, ID number, telephone number;
2. Disease-related information: TNM stage, pathology, histology differentiation, location of tumor, previous treatment history (such as surgery, chemotherapy, TCM), complication (intestinal obstruction);
3. Past history: hypertension, diabetes, smoking history, drinking history, tumor-related family history;
4. Recurrence or metastasis time: first and last follow-up time of the patient, including the censored time or the specific death time.

PFS, as the main endpoint indicator, was defined as the time from the beginning of treatment to the observation of metastasis recurrence or the occurrence of death.

OS, as a secondary endpoint indicator, was defined as the time from the beginning of treatment to the occurrence of death.

### 2.4. Statistical Analysis

Input the clinical data to Epidata 3.1 and create data sources. Data analysis was conducted by SPSS22.0 (IBM, Armonk, New York, USA) and Stata 12.0 (Stata Corporation, College Station, TX, USA). Pearson's *χ*^2^ test was used to examine differences in proportion between TCM group and control group. Life table method was used to calculate the cumulative recurrence and metastasis rates of 1 to 6 years, median progression-free survival time and median overall survival time in each group. Survival analysis was performed by Kaplan-Meier curve, the difference of survival between two groups were calculated by Log-rank test. Cox proportional hazard regression model was established to explore the effect of independent factors on the survival prognosis of the CRC patients. Additionally, PSM was used to perform a 1 : 1 match on fourteen factors of two groups, including age, gender, pathology, stage, histology differentiation, location, surgery, chemotherapy, hypertension, diabetes, smoking history, drinking history, intestinal obstruction and family history. Moreover, the match tolerance of PSM was set at 0.05 and replacement was not allowed. All statistical tests used a two-sided test. *p* value while was less than 0.05 was considered to be statistically significant.

## 3. Results

### 3.1. Baseline Characteristics

As of December 30, 2018, the last follow-up date, a total of 529 patients were included in our retrospective cohort study. Before PSM, there were 285 patients in the TCM group and 244 patients in the control group. There were statistically significant differences between the two groups in factors such as histological differentiation, primary location of tumor, smoking history, intestinal obstruction and TNM stage (*p* < 0.05). After PSM, 175 patients in each group were matched, which leaded to the equilibrium of covariates between the two groups. Baseline characteristics and clinical features before and after PSM were both displayed in Table 1.

### 3.2. Comparison of Survival Time and Survival Rates between the Two Groups

**Figure 2:**
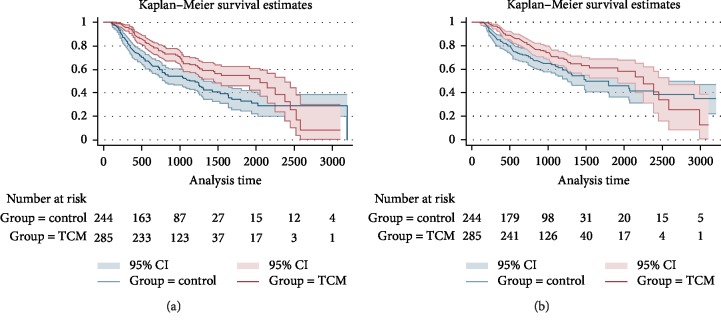
Kaplan-Meier curve of PFS and OS of CRC patients (before PSM). (a) K-M curve of PFS, (b) K-M curve of OS.

Before PSM, the median progression-free survival (mPFS) time of the TCM group and the control group were 68 months and 40 months respectively (HR: 0.608, 95%CI: 0.468 to 0.790, log-rank *p* < 0.001), while the median overall survival (mOS) time were 75 months and 65 months respectively. (HR: 0.713, 95%CI: 0.533 to 0.954, log-rank *p*=0.022), as shown in Figure 2.

**Figure 3:**
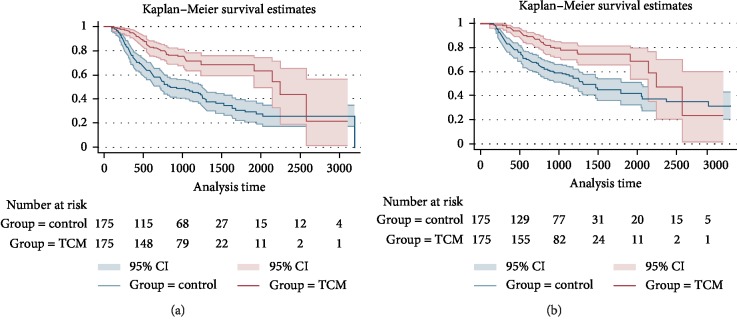
Kaplan-Meier curve of PFS and OS of CRC patients (after PSM). (a) K-M curve of PFS, (b) K-M curve of OS.

After PSM, the median progression-free survival (mPFS) time of the TCM group and the control group were 75 months and 28 months respectively (HR: 0.402, 95%CI: 0.286 to 0.565, log-rank *p* < 0.001), the median overall survival (mOS) time were 75 months and 44 months respectively (HR: 0.431, 0.294 to 0.631, *p* < 0.001), as shown in Figure 3.

**Figure 4:**
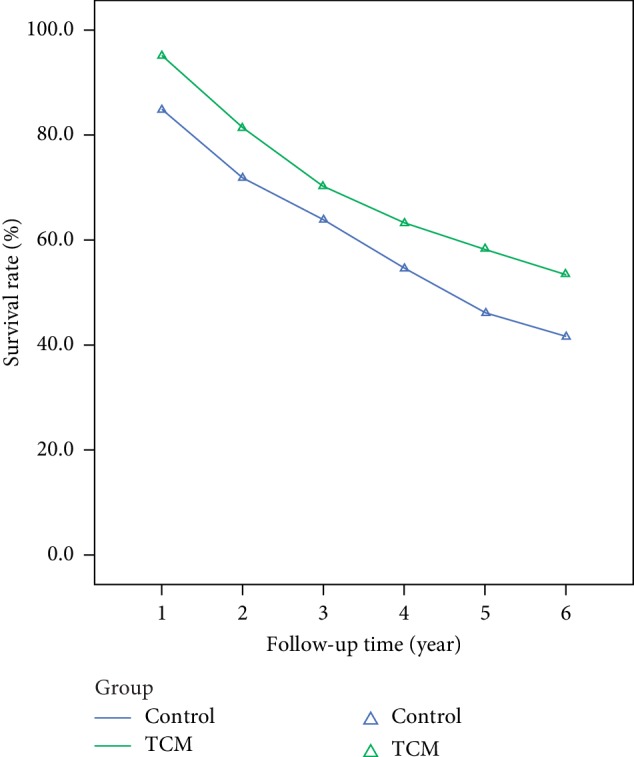
Comparison of overall survival rate of CRC patients.

**Figure 5:**
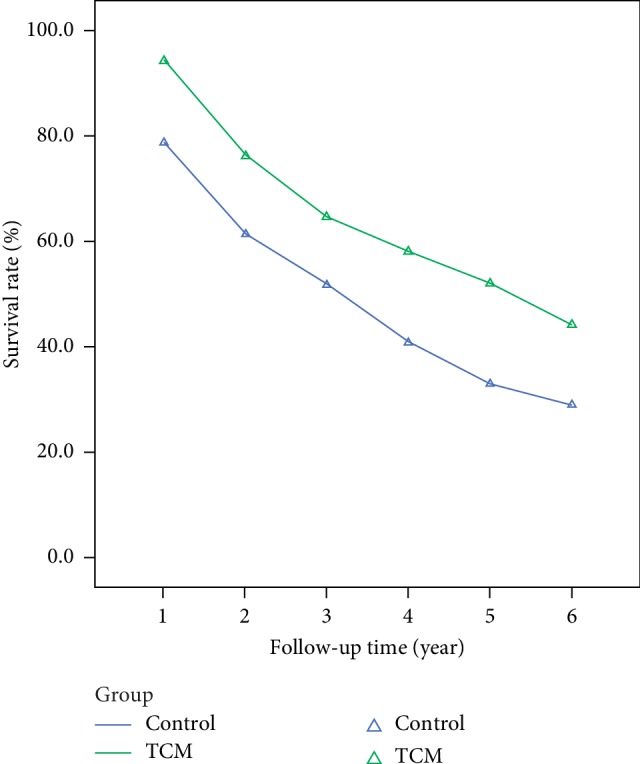
Comparison of progression-free survival rate of CRC patients.

The 1∼6-year overall survival rate and progression-free survival rate of the TCM group were both significantly higher while compared with that of the control group, as shown in Figures 4 and 5 and Table 2.

From the above conclusion, we found that after PSM, the TCM group showed a greater advantage over the control group in improving the survival time of CRC patients.

### 3.3. Cox Regression and Subgroup Analysis of Hazard Ratio (HR)

Cox regression was used to analyze the potential factors which could influence the prognosis of CRC patients. Recurrence and metastasis were defined as the outcome. A total of 15 factors which may have an impact on the progression-free survival time of CRC patients were included.

The result of the Cox regression indicated that surgery, chemotherapy and taking TCM decoction were protective factors for recurrence and metastasis of CRC, while combining with intestinal obstruction, drinking history and family history were independent risk factors. Additionally, tumor stage and histological differentiation also influenced the recurrence of CRC. The earlier the stage, the higher degree of histological differentiation, the less likely tumor relapsed, which was also consistent with clinical practice, as shown in Table 3.

**Figure 6:**
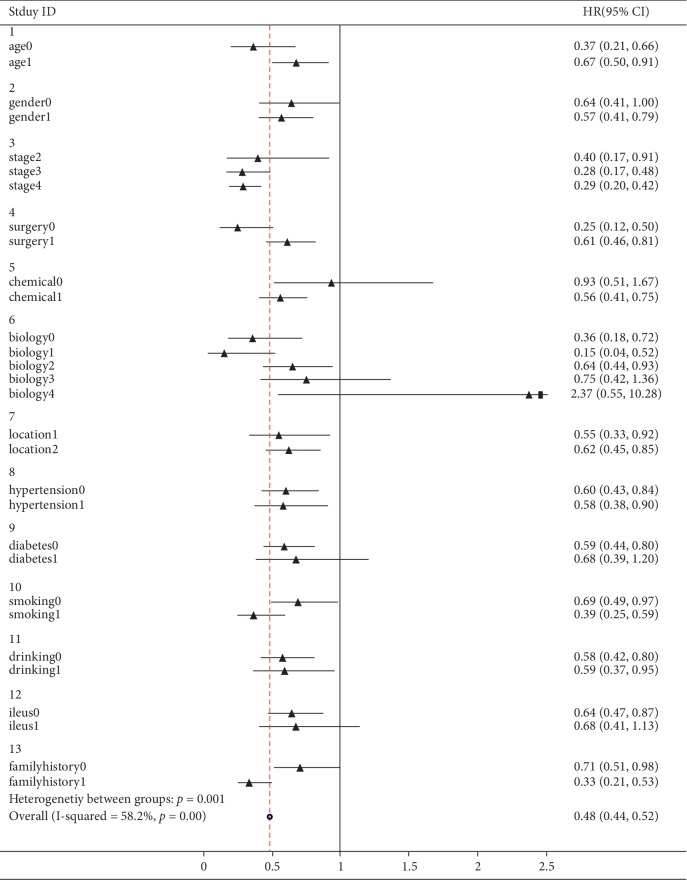
Forest plot of HR and 95% confidence interval between TCM and control group.

The outcome of the subgroup analysis of HR suggested that the HRs of patients in the TCM group were generally lower than the control group patients (HR = 0.48, 95%CI: 0.44–0.52). In addition to this, the HR of patients younger than 60 years old with no surgery, low histological differentiation, smoking and family history in the TCM group was significantly lower than that of the control group, as shown in Figure 6.

### 3.4. Influence of TCM on Survival of CRC in Different Stages

**Figure 7:**
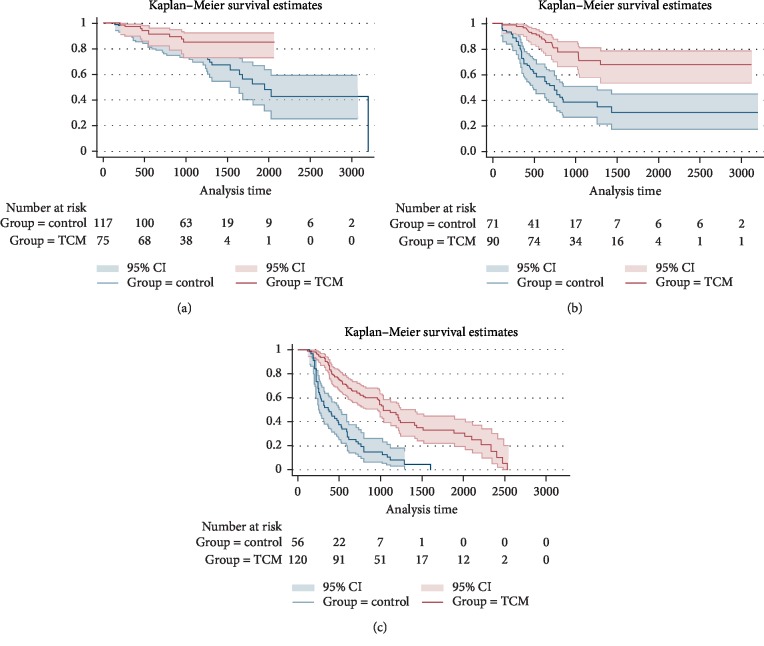
Progression-free survival curves for patients with stage I + II (a), III (b), IV (c) (before PSM).

Based on the results of the above survival analysis, we further explored the effect of TCM treatment on the progression-free survival of CRC at each stage, and it turned out that the mPFS of stage I CRC patients in both groups were not given, there was no statistically significant difference between the two groups (*p* > 0.05). mPFS was 65 months in the stage I + II TCM group and 56 months in the control group, and the difference was statistically significant (*p*=0.023). The survival curve of patients in stage I + II was shown in Figure 7(a). mPFS of stage III patients was 39 months in the TCM group and 25 months in the control group, and the difference was statistically significant (*p* < 0.001), as shown in Figure 7(b). mPFS of stage IV patients was 35 months in the TCM group and 12 months in the control group, and the difference was statistically significant (*p* < 0.001), as shown in Figure 7(c).

**Figure 8:**
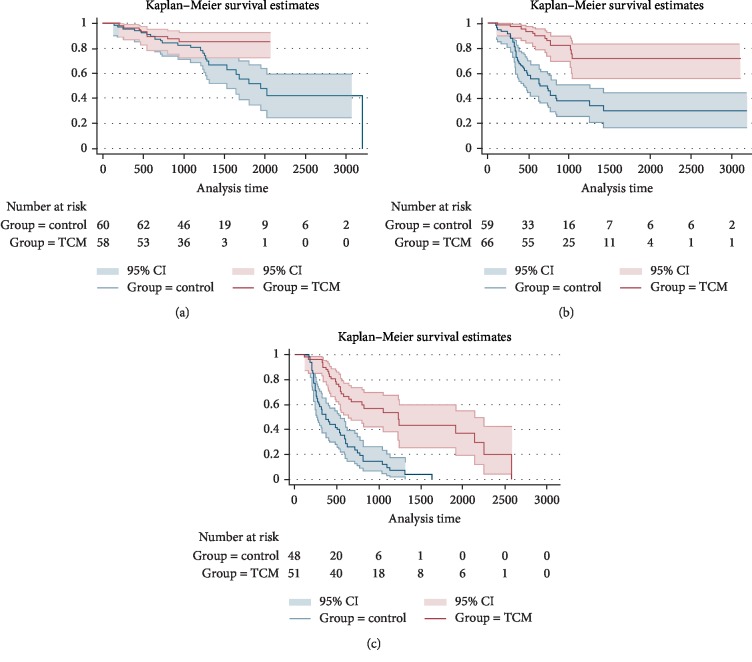
Progression-free survival curves for patients with stage I + II (a), III (b), IV (c) (after PSM).

After PSM, for patients with stage III and IV, we came to the same conclusions that mPFS of the TCM groups were longer than the control group, the difference was statistically significant (*p* < 0.001), as shown in Figures 8(b) and 8(c). Comparing patients of the control group with early stage (I + II), the TCM group showed certain advantages in prolonging the mPFS. However, the difference was not statistically significant (*p* > 0.05), as shown in Figure 8(a).

## 4. Discussion

With the deepening understanding and research of TCM in the world, TCM has increasingly highlighted its unique value in the comprehensive treatment of CRC and demonstrated its advantages of individualized treatment. Compared with the precise treatment mode of modern medicine, TCM can achieve a “seamless” connection with the current clinical treatment methods for CRC by holistic adjustment and its treatment based on syndrome differentiation.

A number of clinical studies showed that the combination of TCM and chemotherapy for CRC is more effective than chemotherapy alone, which can significantly improve the quality of life and prolong the survival time of patients [8, 18–21]. In addition, TCM combined with chemotherapy has a significant effect on preventing postoperative recurrence and metastasis of CRC, alleviating leucopenia after chemotherapy and increasing bone marrow reserve [6, 22]. The combination of TCM and high-frequency hyperthermia has high safety, and is superior to the single treatment in terms of disease control rate, total effective rate and quality of life of advanced CRC [23, 24]. Moreover, TCM also can be combined with surgery. Before surgery, TCM can be used to strengthen healthy Qi, create positive conditions for surgery and facilitate the smooth operation. After the operation, it can cooperate with TCM to regulate spleen and stomach, cultivate the foundation of acquired constitution and promote the recovery of body by replenishing Qi and nourishing Yin. Besides, TCM can also make full preparation for necessary radiotherapy and chemotherapy after surgery. For some patients with advanced rectal cancer who have lost the opportunity of surgery, radiotherapy and chemotherapy, TCM can directly apply drugs to the rectum for external treatment, so that drugs can directly act on the lesion, avoiding the disadvantages of the drug's stimulation on the gastrointestinal mucosa and the first-pass effect in the liver, which plays a positive role in improving the quality of life of patients with advanced CRC [25, 26]. The above literatures have demonstrated from different perspectives that TCM, as an important component of CRC treatment, has prominent advantages in prolonging the survival period of patients, improving the quality of life and reducing the adverse reactions. However, most of the clinical reports were small sample studies with low level of evidence-based medicine. Therefore, our study attempted to deeply explore and demonstrate the efficacy of TCM in the treatment of CRC based on the multi-center and large sample size.

Our study results showed that the application of Anti-cancer Decoction II Formula had a positive effect on the mPFS and mOS of patients with CRC, and reduce the risk of CRC recurrence and metastasis effectively. Considering the imbalanced baseline of clinical data between the two groups, we balanced the covariates between the two groups by PSM. After PSM, long-term TCM treatment still had a huge advantage in improving the survival time of CRC and reducing the risk of recurrence and metastasis. COX multivariate regression analysis also showed that taking Chinese herbal decoction was an independent protective factor which affected the prognosis of patients with CRC. Moreover, surgery and chemotherapy were protective factors for recurrence and metastasis of CRC. Intestinal obstruction, previous alcohol consumption and family history were independent risk factors for recurrence and metastasis. TNM stage and histological differentiation were also closely associated with the prognosis of CRC. Subgroup analysis showed that long-term TCM treatment could significantly reduce the risk of recurrence and metastasis of patients in each subgroup, and the risk ratio of recurrence and metastasis were significantly lower in patients in the TCM treatment group who were younger than 60 years old, no surgery, with low histological differentiation, smoking history and family history.

Through further study on the survival of patients with CRC at each stage, we found that Anti-cancer Decoction II Formula had an improved effect on the survival of patients with CRC at all stages, especially CRC patients with stage III∼IV, and the progression-free survival was significantly higher than that of the control group. Although there was no statistically significant difference in the survival curve of CRC patients with stage I, it may be related to the fewer sample size and end-point events. However, according to the comprehensive analysis of CRC patients with stage I + II, the application of Anti-cancer Decoction II Formula in patients with early stage CRC also had a significant effect on progression-free survival.

Considering some confounding factors while comparing two groups' patients with different stages, we conducted another intergroup comparison of patients in each stage after PSM. Finally, similar conclusions were obtained that CRC patients with stage III ∼ IV benefited from the long-term treatment of TCM in prolonging the mPFS. The clinical effect of patients with early stage was not statistically significant, it may be due to the small sample size of each stage or fewer endpoint events. However, it showed a tendency to improve the survival time, which needed to be verified by more rigorous clinical trials in the future.

Therefore, long-term TCM treatment by the usage of the modified Anti-cancer Decoction II Formula can be flexibly applied in the whole course of CRC treatment, which is beneficial to the improvement of survival time. The specific treatment principle should be based on the principle which Ming dynasty <Yi Zong Bi Du> said, at the beginning of the disease, the healthy Qi is still strong, the pathogenic factors is still shallow, so eliminate pathogens mainly; while patients suffer disease gradually long, the pathogenic factors is deeper, the healthy Qi is weaker, then apply reinforcement and elimination in combination; At the end of the disease, the pathogenic factors is hyperactive while the healthy Qi is weak, so strengthen healthy Qi mainly. By adjusting the proportion of strengthening healthy Qi and eliminating pathogens in herbals, we can flexibly adapt to the intricate changes of the disease. TCM treatment philosophy is in line with the law of multiplicity of tumor etiology, complexity of pathogenesis and comprehensive treatment, which gives full play to the advantages of TCM in anti-tumor.

There were also some deficiencies in our study, such as the number of cases was still insufficient, especially in CRC patients with stage I, as well as the lack of endpoint events, which directly led to the inability to calculate specific statistical data. In addition, the majority of patients included in our study were from Shanghai, China, which had poor representativeness. Secondly, although the method of PSM was adopted to balance the covariates between the two groups, which have eliminated the disadvantages of imbalanced baseline of the original data. However, as a retrospective clinical study, there was a lack of randomness, as well as a number of other defects, such as loss of follow-up bias, information bias, and recall bias. As a result, the evidence level of our research results was still not high and persuasive enough. Therefore, RCTs with well homogeneity will be conducted in the future to provide more sufficient demonstration.

## 5. Conclusion

Long-term TCM treatment by the usage of the modified Anti-cancer Decoction II Formula not only has a positive effect on the survival time of CRC patients, but also helps reduce the risk of recurrence and metastasis of CRC, which can be flexibly applied in the whole process of CRC treatment.

## Figures and Tables

**Table 1:** Baseline characteristics and clinical features of TCM group and the control group before and after PSM.

Group	Before PSM			After PSM		
	TCM *n* = 285	Control *n* = 244	*p* value	TCM *n* = 175	Control *n* = 175	*p* value
Gender						
Male	170 (59.6)	147 (60.2)	0.889	106 (60.6)	102 (58.3)	0.663
Female	115 (40.4)	97 (39.8)		69 (39.4)	73 (41.7)	
Pathology						
None	16 (5.6)	9 (3.7)	0.247	11 (6.3)	8 (4.6)	0.510
Adenocarcinoma	259 (90.9)	226 (92.6)		160 (91.4)	159 (90.9)	
Squamous cell Ca	1 (0.4)	4 (1.6)		1 (0.6)	4 (2.3)	
Others	9 (3.2)	5 (2.0)		3 (1.7)	4 (2.3)	
Stage						
I	17 (6.0)	24 (9.8)	<0.001^*∗*^	14 (8.0)	10 (5.7)	0.538
II	58 (20.4)	93 (38.1)		44 (25.1)	57 (32.6)	
III	90 (31.6)	71 (29.1)		66 (37.7)	60 (34.3)	
IV	120 (42.1)	56 (23.0)		51 (29.1)	48 (27.4)	
Histodifferentiation						
Unknown	40 (14.0)	18 (7.4)	0.005^*∗*^	21 (12.0)	16 (9.1)	0.460
Low	14 (4.9)	8 (3.3)		3 (1.7)	8 (4.6)	
Moderate	179 (62.8)	151 (61.9)		110 (62.9)	97 (55.4)	
Moderate-low	16 (5.6)	47 (19.3)		11 (6.3)	35 (20.0)	
Moderate-high	36 (12.6)	20 (8.2)		30 (17.1)	19 (10.9)	
Location						
Rectum	115 (40.4)	45 (18.4)	＜0.001^*∗*^	43 (24.6)	43 (24.6)	1
Colon	170 (59.6)	199 (81.6)		132 (75.4)	132 (75.4)	
Surgery						
Yes	258 (90.5)	224 (91.8)	0.607	158 (90.3)	160 (91.4)	0.711
No	27 (9.5)	20 (8.2)		17 (9.7)	15 (8.6)	
Chemotherapy						
Yes	221 (77.5)	198 (81.1)	0.309	143 (81.7)	145 (82.9)	0.779
No	64 (22.5)	46 (18.9)		32 (18.3)	30 (17.1)	
Hypertension						
Yes	79 (27.7)	81 (33.2)	0.172	53 (30.3)	50 (28.6)	0.725
No	206 (72.3)	163 (66.8)		122 (69.7)	125 (71.4)	
Diabetes						
Yes	75 (26.3)	65 (26.6)	0.933	47 (26.9)	32 (18.3)	0.055
No	210 (73.7)	179 (73.4)		128 (73.1)	143 (81.7)	
Smoking						
Yes	122 (42.8)	60 (24.6)	＜0.001^*∗*^	56 (32.0)	54 (30.9)	0.818
No	163 (57.2)	184 (75.4)		119 (68.0)	121 (69.1)	
Drinking						
Yes	68 (23.9)	55 (22.5)	0.720	35 (20.0)	36 (20.6)	0.894
No	217 (76.1)	189 (77.5)		140 (80.0)	139 (79.4)	
Intestinal obstruction						
Yes	33 (11.6)	45 (18.4)	0.026^*∗*^	23 (13.1)	31 (17.7)	0.236
No	252 (88.4)	199 (81.6)		152 (86.9)	144 (82.3)	
Family history						
Yes	89 (31.2)	58 (23.8)	0.056	50 (28.6)	47 (26.9)	0.720
No	196 (68.8)	186 (76.2)		125 (71.4)	128 (73.1)	
Age						
>60	220 (77.2)	204 (83.6)	0.065	146 (83.4)	140 (80.0)	0.407
≤60	65 (22.8)	40 (16.4)		29 (16.6)	35 (20.0)	

PSM: propensity scores matching; ^*∗*^*p* < 0.05, there was a significant statistical difference in the subgroup.

**Table 2:** Comparison of progression-free survival of CRC patients [Case (%)].

Group	Cumulative recurrence/survival rate (%)					
	1-year	2-year	3-year	4-year	5-year	6-year
TCM	17/94.0	67/76.1	101/64.7	120/57.9	137/52.0	159/44.1
Control	52/78.6	94/61.4	118/51.7	144/40.8	163/33.0	173/29.1
*χ* ^2^	27.296	13.307	9.048	15.038	18.791	12.845
*p*	<0.01	<0.01	<0.01	<0.01	<0.01	<0.01

**Table 3:** Cox regression results of CRC patients.

Factor	*B*	Wald	*p*	HR	95%CI
TCM	−1.052	42.474	<0.001	0.349	0.255–0.479
Surgery	−0.708	9.382	0.002	0.493	0.313–0.775
Chemotherapy	−0.539	9.544	0.002	0.583	0.414–0.821
Drinking	0.361	5.784	0.016	1.435	1.069–1.927
Intestinal obstruction	0.344	4.380	0.036	1.410	1.022–1.945
Family history	0.467	10.197	0.001	1.596	1.198–2.126
Stage					
I as control	NA	53.702	<0.001	NA	NA
II	−3.754	26.213	<0.001	0.023	0.006–0.099
III	−1.225	30.068	<0.001	0.294	0.189–0.455
IV	−0.720	17.044	<0.001	0.487	0.346–0.685
Histodifferentiation					
Unknown as control	NA	160.669	<0.001	NA	NA
Low	2.496	31.831	<0.001	12.132	5.098–28.871
Moderate	4.449	93.771	<0.001	85.523	34.756–210.447
Moderate-low	2.043	27.511	<0.001	7.716	3.596–16.556
Moderate-high	3.787	80.909	<0.001	44.118	19.331–100.685

## Data Availability

This study is a retrospective cohort study and the data involved is available from the corresponding author upon request and privacy-related parts of the patient will not be provided.

## Abbreviations

TCM: Traditional Chinese medicine
CRC: Colorectal cancer
PSM: Propensity score matching
PFS: Progression-free survival
OS: Overall survival
HR: Hazard ratio
